# Three-year surveillance of myopia trends and urban–rural disparities among schoolchildren in a Coastal City of Southern China

**DOI:** 10.3389/fpubh.2026.1878486

**Published:** 2026-07-24

**Authors:** Wenting Hou, Tianfeng Lai, Qiuling Pan, Fangjiao Tang, Dongling Chen, Zhijie Shen, Junhui Fu, Jiemin Su, Linyan Zhang, Qi Chen, Xianxian Wei

**Affiliations:** 1 Department of Optometry, the Second People’s Hospital of Qinzhou, Qinzhou, China; 2 Visual Science and Optometry Center, the People’s Hospital of Guangxi Zhuang Autonomous Region, Nanning, China

**Keywords:** adolescents, China, myopia, repeated cross-sectional study, school-based screening, urban–rural disparities

## Abstract

**Objective:**

Myopia has become an increasingly important vision health concern among children and adolescents in China. However, longitudinal surveillance data from less-developed coastal regions remain limited. This study aimed to investigate temporal trends and demographic differences in myopia prevalence among school students in Qinzhou, Southern China, using three consecutive years of school-based screening data.

**Methods:**

A repeated cross-sectional study was conducted from 2022 to 2024 in 40 primary and secondary schools in Qinzhou, China. A total of 90,072 screening records from students aged 6–18 years were included. Uncorrected visual acuity and non-cycloplegic autorefraction were assessed using standardized examination procedures. Myopia was defined according to spherical equivalent refraction. Differences in myopia prevalence by sex, educational stage, residential region, and age group were analyzed using chi-square tests and binary logistic regression models.

**Results:**

Overall myopia prevalence increased from 43.17% in 2022 to 48.21% in 2023 and remained high in 2024 (47.51%). Female students consistently showed higher myopia prevalence than male students across all years (all *p* < 0.001). Myopia prevalence increased significantly with advancing educational stage and age, particularly after 12 years of age. Within matched educational stages, urban students demonstrated persistently high prevalence rates, whereas larger year-to-year fluctuations were observed among rural and township students. In addition, the proportions of moderate and high myopia increased with age. Although a slight decrease in prevalence was observed in 2024, the overall burden of myopia remained substantial.

**Conclusion:**

Myopia prevalence among school-aged children and adolescents in Qinzhou remained high during the study period, with significant disparities across sex, age, educational stage, and residential region. These findings highlight the need for early preventive interventions and region-specific myopia control strategies, particularly for younger students and underserved populations.

## Introduction

Myopia is one of the most common visual impairments worldwide, and its prevalence has been steadily increasing in recent years, posing a major public health concern. According to the World Health Organization (WHO), over 2 billion people worldwide are currently myopic, and this number may rise to 5 billion by 2050 (1, 2). In China, the school student myopia rate reached 52.7% in 2022, indicating the continued severity of the issue.

Myopia not only affects visual quality and daily life but can also lead to severe ocular complications such as retinal detachment, cataracts, and even irreversible vision loss or blindness (3, 4). Therefore, early screening and timely intervention are essential to delay myopia progression and reduce the risk of associated complications (5, 6).

With growing attention to myopia prevention and control in China, many regions have implemented large-scale myopia screening programs (7–11). However, most published epidemiological data originate from economically developed metropolitan areas such as Beijing, Shanghai, and Guangzhou, where healthcare resources and public health infrastructure are relatively advanced. In contrast, less-developed prefecture-level coastal cities in Southern China remain underrepresented in the literature, despite potentially having distinct demographic structures, urban–rural disparities, educational pressures, and healthcare accessibility. These contextual differences may influence the distribution and progression of myopia among school-aged children.

Qinzhou, located in Guangxi Zhuang Autonomous Region, is a rapidly developing coastal city characterized by a mixed urban–rural population and varying socioeconomic conditions. As an important node in regional economic development, Qinzhou has experienced significant educational expansion and lifestyle transitions in recent years, which may affect visual health patterns among children and adolescents. Nevertheless, comprehensive and continuous surveillance data on myopia prevalence in this region are scarce. Generating region-specific epidemiological evidence is crucial for tailoring prevention strategies to local conditions and optimizing allocation of public health resources.

Furthermore, while previous studies have primarily reported cross-sectional prevalence rates, limited research has examined multi-year trends within the same municipal screening framework, particularly with stratified comparisons between urban and rural schools. The present study analyzes three consecutive years (2022–2024) of large-scale school-based screening data in Qinzhou to evaluate temporal trends in myopia prevalence and explore demographic disparities. By providing repeated cross-sectional, real-world surveillance data from an underreported region, this study aims to supplement existing evidence and provide a scientific basis for region-specific myopia prevention strategies.

## Participants and methods

### Participants

With support from the Qinzhou Health Commission, the Department of Optometry at the Second People’s Hospital of Qinzhou conducted annual myopia screenings from September 2022 to December 2024 in 40 schools, including 32 primary and 8 secondary schools.

The recruitment process was implemented in a stepwise manner. First, the Qinzhou Health Commission coordinated with the Municipal Education Bureau to notify eligible schools about the annual vision screening program. After obtaining agreement from school administrators, an official notice describing the purpose, procedures, and significance of the screening was distributed to students and their parents or legal guardians at least 1 week prior to the scheduled examination. Written informed consent forms were attached to the notice and required to be signed by parents or legal guardians for students under 16 years of age. Only students who returned signed consent forms were included in the screening. Students who were absent on the examination day or failed to provide signed consent were excluded from the study.

All examinations were conducted on school premises during regular school hours by trained optometrists following a standardized protocol. This study was approved by the Ethics Committee of the Second People’s Hospital of Qinzhou (No: 2023–3-234). Informed consent was obtained from all participants as well as from the parents or legal guardians of all participants under the age of 16. This study was conducted in accordance with the Declaration of Helsinki.

### Methods

Screenings included uncorrected visual acuity (UCVA) and non-cycloplegic autorefraction (12). The study was based on the annual government-led school vision surveillance program in Qinzhou. A cluster-based design was adopted, with schools serving as the sampling units. A total of 40 primary and secondary schools were included, representing all public schools participating in the municipal screening program during the study period. Therefore, this study reflects population-based surveillance data rather than a sample survey, and no additional sample size calculation was performed. The coverage rate of eligible students exceeded 95%, ensuring strong representativeness of the local school-aged population.

Students wearing orthokeratology lenses were asked to stop lens use for 1 week, which is a commonly adopted washout period in epidemiological studies and is generally sufficient for most corneal reshaping effects to regress, allowing refractive status to return close to baseline levels (13). UCVA was measured using a digital LCD eye chart (Beijng Eyenurse Technology Co., Ltd) at a 5-meter distance, in accordance with national standards (GB11533-2011) (14). The visual acuity chart was wirelessly connected to a smartphone via Bluetooth, allowing the examiner to control optotype presentation through a dedicated mobile application. During testing, optotypes were displayed sequentially according to the standardized logarithmic progression. Participants were instructed to identify the direction of the optotype opening, and examiners recorded responses in real time using the mobile interface. Each optotype was judged as correct or incorrect based on the participant’s verbal or gestural feedback, and visual acuity was determined according to standard stopping criteria defined by the national protocol.

Autorefraction was performed with a desktop autorefractor (NIDEK AR-1), with three measurements taken and averaged. Spherical equivalent (SE = sphere +1/2 × cylinder) was used to determine refractive status, with the worse eye recorded if results differed (15, 16). Myopia severity was classified as: low myopia (−3.00D ≤ SE < −0.50D), moderate myopia (−6.00D ≤ SE < −3.00D), and high myopia (SE < −6.00D). All examiners were trained optometrists who received standardized training prior to data collection to ensure consistency and reliability.

### Data collection and quality control

All screenings were performed by trained professionals from the hospital. Data were uploaded to a centralized monitoring system. A random 5% of students were re-evaluated for quality assurance. All reported cases represent screening-detected myopia.

### Statistical analysis

Descriptive statistics were calculated using Python 13.1. Myopia prevalence was expressed as *n* (%), and continuous variables were reported as mean ± standard ( $\bar{x}\pm s$ ) deviation. Chi-square tests compared differences between groups. Binary logistic regression was used to assess prevalence trends over time. Odds ratios (ORs) and *p*-values were calculated, with *p* < 0.05 considered statistically significant.

## Results

### General characteristics

A total of 90,072 screening records from students aged 6 to 18 years were analyzed. The numbers of students screened and their demographic composition from 2022 to 2024 are detailed in Table 1.

**Table 1:** Student numbers and gender distribution by education level (2022–2024).

Category	2022	2023	2024
Total	31,009	29,737	29,326
Male [*n* (%)]	16,526 (53.29)	15,716 (52.85)	15,426 (52.60)
Female [*n* (%)]	14,483 (46.71)	14,021 (47.15)	13,900 (47.40)
Age [ $\bar{x}\pm s$ ]	10.77 ± 2.91	11.12 ± 2.87	11.12 ± 2.92
Primary school [*n* (%)]	19,547 (63.04)	17,702 (59.53)	16,801 (57.29)
Secondary school [*n* (%)]	9,097 (29.34)	9,359 (31.47)	9,975 (34.01)
High school [*n* (%)]	2,365 (7.63)	2,676 (9.00)	2,550 (8.70)

n, number of students.

### Trends in myopia prevalence

**Figure 1:**
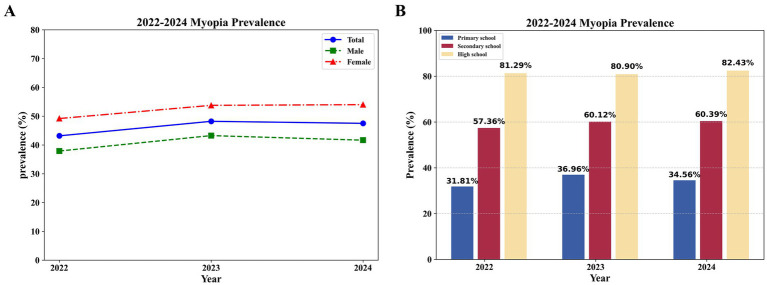
Changes in myopia prevalence by demographic characteristics, 2022–2024: **(A)** Sex; **(B)** education level.

Myopia prevalence showed fluctuations over the three-year period: 43.17% in 2022, increasing to 48.21% in 2023, and slightly decreasing to 47.51% in 2024. The average UCVA remained relatively stable across years (4.82 ± 0.31 in 2022, 4.77 ± 0.34 in both 2023 and 2024), indicating consistent screening quality. In summary, while the myopia rate rose in 2023, it experienced a modest rebound in 2024 (Table 2, Figure 1A).

**Table 2:** Vision and myopia rates (2022–2024).

Category	2022	2023	2024
Total [*n*]	31,009	29,737	29,326
UCVA [mean ± SD]	4.82 ± 0.31	4.77 ± 0.34	4.77 ± 0.34
Myopia [*n* (%)]	13,387 (43.17)	14,335 (48.21)	13,932 (47.51)

n, number of students.

### Comparison of myopia prevalence between genders

Based on data from 2022 to 2024, both male and female students exhibited a year-on-year increase in myopia prevalence, with statistically significant gender differences in each year (*p* < 0.01). Chi-square tests confirmed a significant impact of gender on myopia prevalence across the 3 years (all *p* < 0.001). Overall, females consistently showed higher myopia rates than males, and the gender gap remained significant from 2022 to 2024 (Table 3 and Figure 1A).

**Table 3:** Myopia rates by sex (2022–2024).

Gender	2022			2023			2024		
	Total	Myopia [*n* (%)]	UCVA [mean ± SD]	Total	Myopia [*n* (%)]	UCVA [mean ± SD]	Total	Myopia [*n* (%)]	UCVA [mean ± SD]
male	16,526	6,264 (37.90)	4.84 ± 0.29	15,716	6,797 (43.25)	4.80 ± 0.32	15,426	6,426 (41.66)	4.80 ± 0.32
female	14,483	7,123 (49.18)	4.77 ± 0.33	14,021	7,538 (53.76)	4.72 ± 0.36	13,900	7,506 (54.00)	4.71 ± 0.36
*χ^2^*		399.70			327.61			446.19	
*p*		<0.001			<0.001			<0.001	

n, number of students.

### Comparison of myopia prevalence across school stages

Based on the analysis of myopia prevalence among primary, junior high, and senior high school students from 2022 to 2024, significant increases were observed in primary and junior high school stages over the three-year period, with changes reaching statistical significances (*p* < 0.001). Conversely, the myopia rate among senior high school students showed minimal variation, with no statistically significant difference (*p* = 0.242), remaining at a consistently high level (approximately 80%). Overall, the upward trend in the rate remained relatively stable at the senior high school level (Table 4 and Figure 1B).

**Table 4:** Myopia rates by education level (2022–2024).

Grades	2022			2023			2024		
	Total	Myopia [*n* (%)]	UCVA [mean ± SD]	Total	Myopia [*n* (%)]	UCVA [mean ± SD]	Total	Myopia [*n* (%)]	UCVA [mean ± SD]
Primary school	19,547	6,217 (31.81)	4.90 ± 0.23	17,702	6,543 (36.96)	4.86 ± 0.27	16,801	5,806 (34.56)	4.86 ± 0.27
Secondary school	9,097	5,219 (57.36)	4.72 ± 0.35	9,359	5,627 (60.12)	4.70 ± 0.36	9,975	6,024 (60.39)	4.68 ± 0.37
High school	2,365	1951 (81.29)	4.45 ± 0.41	2,676	2,165 (80.90)	4.44 ± 0.43	2,550	2,102 (82.43)	4.40 ± 0.43
*χ^2^*		3467.49			2574.72			3040.94	
*p*		<0.001			<0.001			<0.001	

n, number of students.

### Comparison of myopia prevalence between urban and rural students

**Figure 2:**
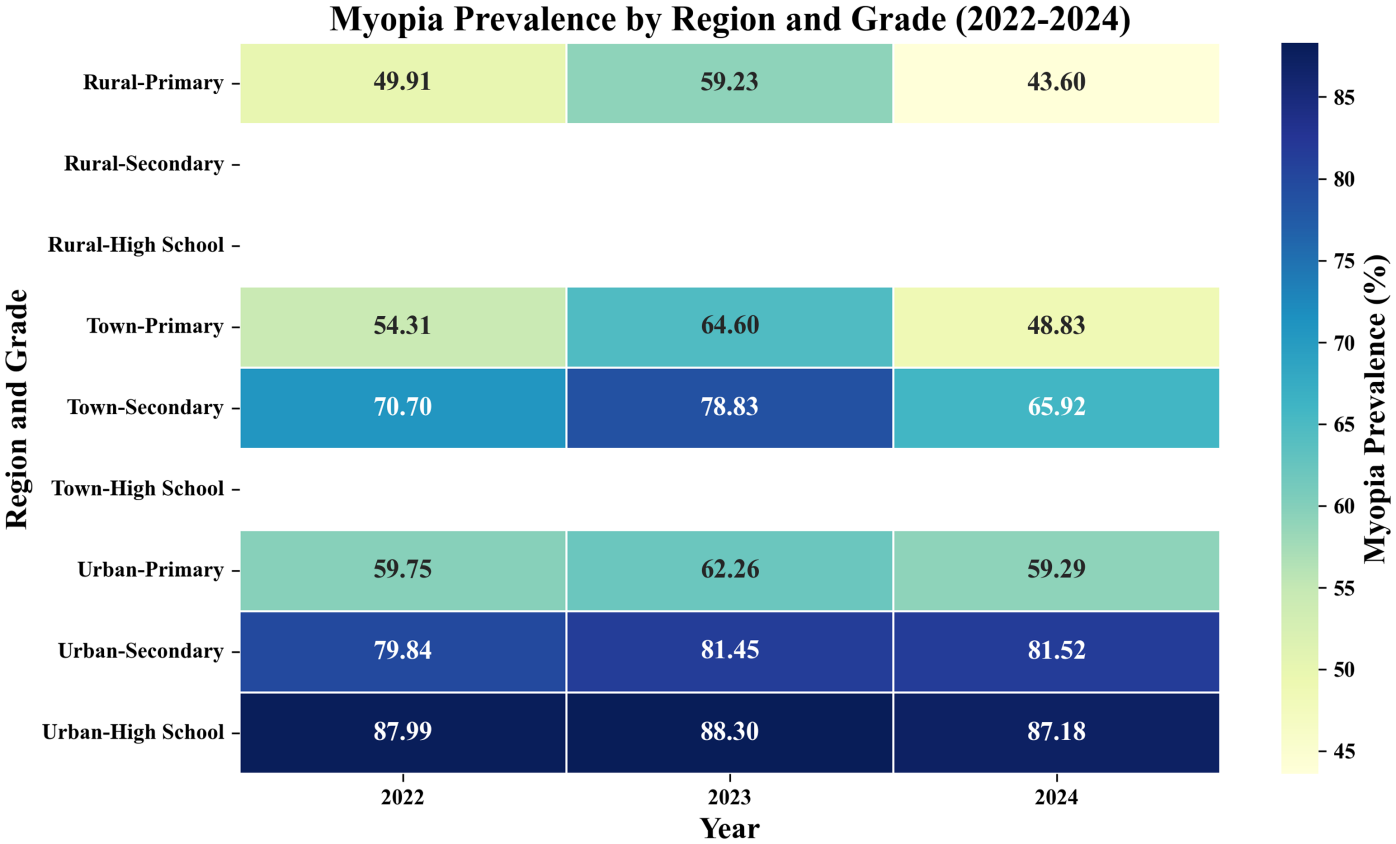
Changes in myopia rates among students in different regions from 2022 to 2024.

Because grade compositions varied across regions, cross-regional comparative analyses were strictly limited to matched educational stages (Table 5). Among primary school students, myopia prevalence was highest in urban areas, followed by towns, and lowest in rural areas. Similarly, among secondary school students, urban areas exhibited a higher prevalence compared to towns. Rural primary school students showed notable fluctuations. Rural primary school students showed notable fluctuations, increasing from 49.91% in 2022 to 59.23% in 2023, before declining to 43.60% in 2024. A similar trend was observed in township primary and junior high school students, whereas urban students maintained relatively stable and consistently high myopia rates, particularly in junior and senior high school, where prevalence remained above 79 and 87%, respectively. In addition, UCVA decreased progressively with advancing school stage across all regions. Overall, changes in myopia prevalence were more pronounced in rural and township areas, while urban areas showed greater stability but higher overall prevalence (Table 5 and Figure 2). It should be noted that in the study region, secondary and high schools are centralized in urban areas; therefore, screening data for rural schools at these educational levels are inherently unavailable.

**Table 5:** Myopia rates by region (2022–2024).

Region	2022			2023			2024		
	Total	Myopia [*n* (%)]	UCVA [mean ± SD]	Total	Myopia [*n* (%)]	UCVA [mean ± SD]	Total	Myopia [*n* (%)]	UCVA [mean ± SD]
Rural area	2,334	1,165 (49.91)		1994	1,181 (59.23)		1750	763 (43.60)	
Primary school	2,334	1,165 (49.91)	4.90 ± 0.18	1994	1,181 (59.23)	4.93 ± 0.18	1750	763 (43.60)	4.92 ± 0.19
Secondary school	–	–		–	–		–	–	
High school	–	–		–	–		–	–	
Town	7,855	5,094 (64.85)		5,938	4,309 (72.57)		7,194	4,319 (60.04)	
Primary school	2,806	1,524 (54.31)	4.92 ± 0.19	2,613	1,688 (64.60)	4.90 ± 0.22	2,478	1,210 (48.83)	4.89 ± 0.23
Secondary school	5,049	3,570 (70.70)	4.79 ± 0.32	3,325	2,621 (78.83)	4.75 ± 0.33	4,716	3,109 (65.92)	4.75 ± 0.34
High school	–	–		–	–		–	–	
Urban area	19,156	12,927 (67.48)		18,759	13,241 (70.56)		17,208	11,692 (67.95)	
Primary school	12,743	7,614 (59.75)	4.89 ± 0.24	11,589	7,215 (62.26)	4.83 ± 0.29	11,157	6,615 (59.29)	4.84 ± 0.28
Secondary school	4,048	3,232 (79.84)	4.64 ± 0.38	4,470	3,641 (81.45)	4.63 ± 0.38	3,501	2,854 (81.52)	4.58 ± 0.41
High school	2,365	2081 (87.99)	4.45 ± 0.41	2,676	2,363 (88.30)	4.44 ± 0.43	2,550	2,223 (87.18)	4.40 ± 0.43

n, number of students. “–” indicates no data. In this region, secondary and high schools are centralized in urban areas and are not present in rural areas.

### Comparison of myopia prevalence by age group

**Figure 3:**
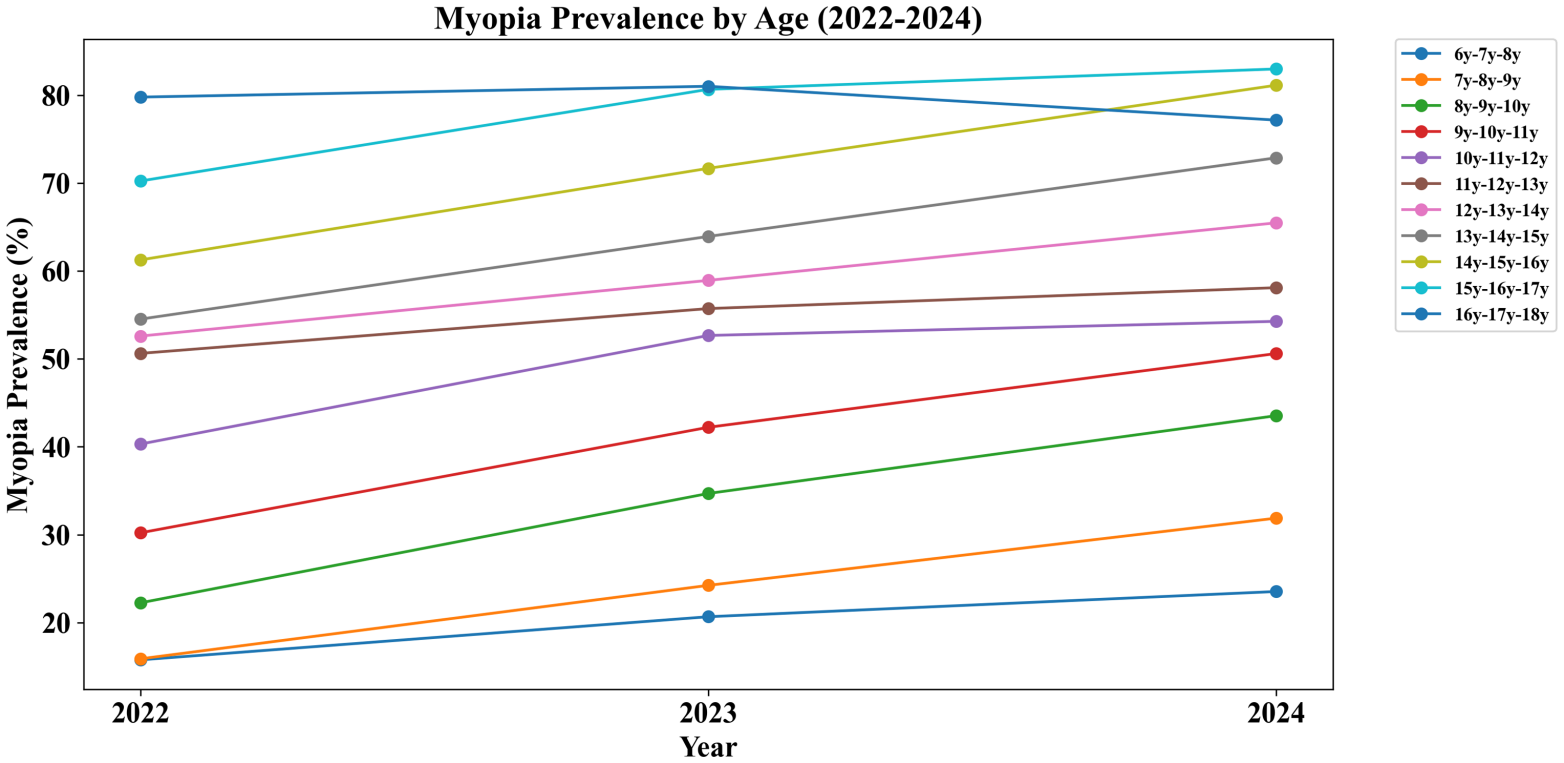
Changes in myopia rates among different age groups from 2022 to 2024.

From 2022 to 2024, myopia prevalence increased significantly with age, ranging from 15.75% in the 6-year-old group to 86.32% in the 18-year-old group—a trend that remained consistent across the 3 years. Between 2022 and 2023, the increase in myopia prevalence was statistically significant for the 6-year-olds (OR = 1.22, *p* = 0.04), 7-year-olds (OR = 1.34, *p* < 0.001), 9-year-olds (OR = 1.23, *p* < 0.001), 12-year-olds (OR = 1.13, *p* = 0.01), and 13-year-olds (OR = 1.19, *p* < 0.001). In contrast, from 2023 to 2024, only the 9-year-old group showed a statistically significant decrease (OR = 0.89, *p* = 0.03). Overall, age groups 6–13 exhibited greater year-to-year variability, while those aged 15 and above had myopia rates consistently over 70% with no significant fluctuation, suggesting stabilization (Table 6 and Figure 3).

**Table 6:** Myopia rates and trends by age group (2022–2024).

Age (y)	2022		2023		2024		2022 vs. 2023		2023 vs. 2024	
	Myopia [*n* (%)]	UCVA [mean ± SD]	Myopia [*n* (%)]	UCVA [mean ± SD]	Myopia [*n* (%)]	UCVA [mean ± SD]	OR	*p*	OR	*p*
6	307 (15.75)	4.97 ± 0.11	251 (18.65)	4.95 ± 0.13	248 (15.71)	4.94 ± 0.14	1.22	0.04*	0.84	0.09
7	447 (15.87)	4.97 ± 0.12	470 (20.66)	4.95 ± 0.14	473 (19.24)	4.94 ± 0.15	1.34	0.00^*^	0.95	0.52
8	728 (22.24)	4.95 ± 0.16	657 (24.23)	4.93 ± 0.17	597 (23.53)	4.93 ± 0.18	1.1	0.14	0.99	0.83
9	1,043 (30.21)	4.92 ± 0.21	1,081 (34.69)	4.89 ± 0.23	867 (31.88)	4.89 ± 0.23	1.23	0.00^*^	0.89	0.03*
10	1,442 (40.31)	4.87 ± 0.26	1,375 (42.22)	4.83 ± 0.29	1,330 (43.54)	4.82 ± 0.29	1.06	0.22	1.07	0.16
11	1728 (50.63)	4.80 ± 0.30	1792 (52.67)	4.76 ± 0.34	1,616 (50.61)	4.76 ± 0.35	1.06	0.20	0.94	0.25
12	1,681 (52.60)	4.77 ± 0.32	2,116 (55.73)	4.73 ± 0.35	1951 (54.28)	4.73 ± 0.35	1.13	0.01*	0.94	0.20
13	1721 (54.53)	4.75 ± 0.33	1995 (58.94)	4.71 ± 0.36	2089 (58.11)	4.70 ± 0.37	1.19	0.00*	0.97	0.56
14	1,669 (61.27)	4.68 ± 0.37	1720 (63.94)	4.66 ± 0.37	1913 (65.49)	4.65 ± 0.37	1.11	0.06	1.07	0.24
15	1,223 (70.25)	4.59 ± 0.40	1,256 (71.69)	4.57 ± 0.41	1,226 (72.89)	4.56 ± 0.40	1.06	0.43	1.06	0.47
16	715 (79.80)	4.48 ± 0.41	856 (80.68)	4.44 ± 0.42	848 (81.15)	4.40 ± 0.42	1.08	0.52	1.04	0.70
17	481(82.93)	4.43 ± 0.41	564 (81.03)	4.44 ± 0.44	615 (83.00)	4.40 ± 0.44	0.86	0.29	1.15	0.30
18	202 (86.32)	4.38 ± 0.42	202 (81.12)	4.45 ± 0.44	159 (77.18)	4.44 ± 0.44	0.71	0.16	0.75	0.20

*p < 0.05, OR: odds ratio.

### Distribution of myopia severity

**Figure 4:**
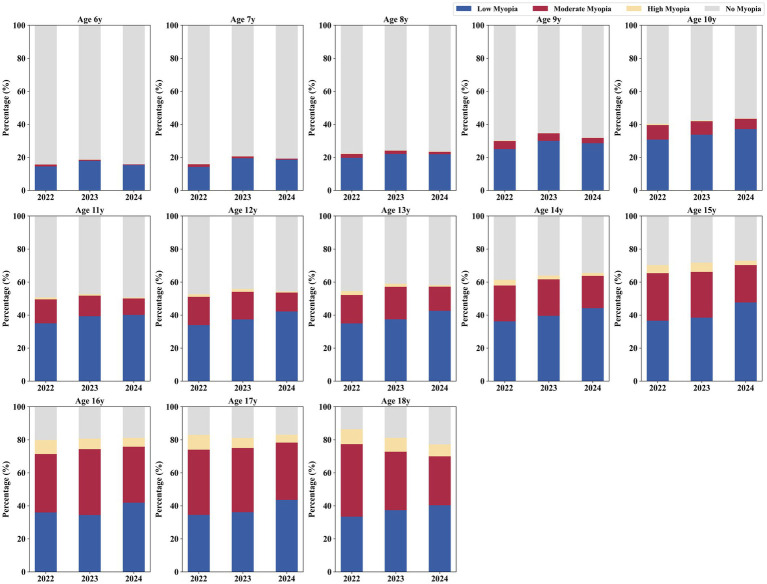
Changes in myopia rates of different severities across age from 2022 to 2024.

The data reveal an age-dependent pattern in myopia severity. With increasing age, the proportion of low myopia progressively rises, while moderate and high myopia show considerable variability across different age groups. In children aged 6–7 years, low myopia predominates; however, from age 12 onward, there is a marked increase in moderate and high myopia. Statistical analysis across 2022–2024 indicates significant changes in the distribution of myopia severity: low (*χ^2^* = 332.65, *p* < 0.001), moderate (*χ^2^* = 104.13, *p* < 0.001), and high myopia (*χ^2^* = 64.09, *p* < 0.001). Similar age-related severity trends have been reported in other large-scale pediatric studies, where mild myopia increases with age while moderate and high myopia show age-sensitive fluctuations (Table 7 and Figure 4).

**Table 7:** Myopia severity by age (2022–2024).

Age (y)	2022			2023			2024		
	Low myopia [*n* (%)]	Moderate myopia [*n* (%)]	High myopia [*n* (%)]	Low myopia [*n* (%)]	Moderate myopia [*n* (%)]	High myopia [*n* (%)]	Low myopia [*n* (%)]	Moderate myopia [*n* (%)]	High myopia [*n* (%)]
6	281 (14.42)	23 (1.18)	3 (0.15)	241 (17.90)	8 (0.59)	2 (0.15)	242 (15.33)	6 (0.38)	0 (0.00)
7	398 (14.13)	47 (1.67)	2 (0.07)	443 (19.47)	24 (1.05)	3 (0.13)	457 (18.59)	15 (0.61)	1 (0.04)
8	644 (19.68)	76 (2.32)	8 (0.24)	599 (22.10)	55 (2.03%)	3 (0.11)	555 (21.88)	37 (1.46)	5 (0.20)
9	861 (24.93)	171 (4.95)	11 (0.32)	933 (29.94)	143 (4.59)	5 (0.16)	776 (28.53)	87 (3.20)	4 (0.15)
10	1,099 (30.72)	313 (8.75)	30 (0.84)	1,096 (33.65)	266 (8.17)	13 (0.40)	1,134 (37.12)	190 (6.22)	6 (0.20)
11	1,195 (35.01)	492 (14.42)	41 (1.20)	1,336 (39.27)	423 (12.43)	33 (0.97)	1,279 (40.06)	318 (9.96)	19 (0.60)
12	1,084 (33.92)	546 (17.08)	51 (1.60)	1,418 (37.35)	633 (16.67)	65 (1.71)	1,514 (42.13)	413 (11.49)	24 (0.67)
13	1,103 (34.95)	541 (17.14)	77 (2.44)	1,267 (37.43)	665 (19.65)	63 (1.86)	1,530 (42.56)	526 (14.63)	33 (0.92)
14	986 (36.20)	590 (21.66)	93 (3.41)	1,063 (39.52)	595 (22.12)	62 (2.30)	1,290 (44.16)	572 (19.58)	51 (1.75)
15	636 (36.53)	501 (28.78)	86 (4.94)	673 (38.41)	485 (27.68)	98 (5.59)	800 (47.56)	382 (22.71)	44 (2.62)
16	322 (35.94)	317 (35.38)	76 (8.48)	365 (34.40)	423 (39.87)	68 (6.41)	437 (41.82)	355 (33.97)	56 (5.36)
17	200 (34.48)	229 (39.48)	52 (8.97)	251 (36.06)	271 (38.94)	42 (6.03)	323 (41.82)	257 (34.68)	35 (4.72)
18	78 (33.33)	103 (44.02)	21 (8.97)	93 (37.35)	88 (35.34)	21 (8.43)	83 (40.29)	61 (29.61)	15 (7.28)
Total	8,887 (28.66)	3,949 (12.74)	551 (1.78)	9,778 (32.88)	4,079 (13.72)	478 (1.61)	10,420 (35.53)	3,219 (10.98)	293 (1.00)

n, number of students; y, year.

## Discussion

Our study demonstrated that from 2022 to 2024, school student myopia prevalences increased overall, peaking at 48.21% in 2023 before slightly declining in 2024. This pattern suggests that myopia issue among youths continues to worsen, likely associated with factors such as intensified academic pressure, increased electronic device usage, and environmental influences. Notably, the years 2022–2023 coincided with the COVID-19 pandemic, during which students experienced extended periods of online learning and confinement at home. This led to a marked increase in visual strain and likely contributed significantly to the rise in myopia prevalence (17–20). The slight rebound observed in 2024 may be attributed to a return to outdoor activities following the easing of pandemic restrictions, the implementation of myopia control measures, and heightened awareness of eye health among parents.

Over the three-year study period, the myopia prevalence among female students consistently exceeded that of males-rising from 49.18 to 54.00% in girls, compared to an increase from 37.90 to 41.66% in boys. This disparity may be attributable to behavioral and physiological factors (21–24). Additionally, hormonal differences may contribute to the higher myopia rates observed in female students (25).

In the comparison of myopia rates across school stages, both primary and junior high students exhibited a noticeable increase. Specifically, the prevalence among primary school students rose from 31.81% in 2022 to 34.56% in 2024, while junior high school students showed an increase from 57.36 to 60.39%. This change may be attributed to several factors: students in these age groups are undergoing rapid growth and development, during which myopia progression tends to accelerate. Additionally, the widespread use of electronic devices, increased academic pressure, and reduced outdoor activity may contribute to this trend (26). In contrast, the myopia rate among senior high school students changed little, likely because their myopia progression had already reached a more stable or slower phase.

Our study focuses on Qinzhou, a coastal city in Guangxi with a mid-level economy, lagging first-tier cities in education, healthcare, and public health awareness. We observed notable disparities in myopia prevalence across different regions when comparing matched educational stages. Myopia rates among primary and secondary students in rural and township areas showed larger fluctuations, whereas those in urban areas exhibited relative stability. When strictly comparing matched primary and secondary school cohorts, students in more affluent urban areas exhibited higher myopia rates than their rural and township counterparts. This disparity may be due to increased exposure to electronic devices, heavier academic workloads, and reduced outdoor activity among urban children (27–29). Simultaneously, rising access to electronic devices in rural areas and inadequate school-based vision care services likely contribute to the increasing myopia rates among rural students (30). Notably, rural primary school students showed a marked increase in myopia prevalence from 49.91% in 2022 to 59.23% in 2023, followed by a substantial decline to 43.60% in 2024. This fluctuation may be related to increased screen exposure and reduced outdoor activity during the late COVID-19 period, particularly in 2023. The decline in overall prevalence observed in 2024 reflects the dynamic nature of repeated cross-sectional data. Older student cohorts graduate and are replaced by incoming cohorts. The normalization of educational routines and outdoor activities in 2024 likely resulted in a lower myopia burden among new students. This demographic turnover reduced the population-level prevalence, even though individual myopia remains irreversible. These findings suggest that environmental and behavioral changes may have a significant impact on myopia development, especially among younger students in rural areas. Similarly, Zhang et al. (31) conducted a school-based vision screening of 173,627 primary school students in three districts of Guangzhou (Yuexiu, Panyu, and Huadu) and found an overall myopia prevalence of 36.97%, with significant inter-district variation (37.67, 32.56, and 38.92%, respectively) (31). Additionally, similar school-based screenings using non-cycloplegic autorefraction in other Chinese regions, such as Wenzhou (10) and Chongqing (8), reported varying prevalence rates, further demonstrating that geographic and socioeconomic variations significantly shape the nationwide epidemiological landscape of childhood myopia (10, 11).

Out data indicate that myopia prevalence increases steadily with age, with a particularly marked rise in individuals aged 12 years and older. Children aged 6 to 12 constitute a critical window for myopia development, implying that prevention efforts should begin at the early primary school stages (32, 33). Moreover, consistent with our findings, Xiao et al. (34) reported that in Shekou district of Shenzhen, the severity of myopia and axial length increased progressively with age and grade level, with moderate and high myopia becoming significantly more common in older students. This aligns with our observation that while low myopia generally increases with age, moderate and high myopia escalate sharply after age 12, highlighting that myopia not only initiates early but also progresses rapidly thereafter, underscoring the necessity for early intervention to prevent high refractive errors and associated ocular complications (35–37).

This study has several limitations. First, it covered only selected schools in Qinzhou, which restricts the representativeness of the sample. Second, we did not perform in-depth analyses of individual factors such as genetic predisposition and lifestyle behaviors, which are known to influence myopia development. Third, the dataset spans only 3 years and includes a relatively small number of senior high school students, which may affect the comprehensiveness of the findings and limit our ability to assess long-term trends. Fourth, refractive error was measured using non-cycloplegic autorefraction rather than cycloplegic refraction, which is considered the gold standard for epidemiological studies in children. The absence of cycloplegia may lead to accommodation-induced measurement bias and a potential overestimation of myopia prevalence, particularly among younger children who typically exhibit stronger accommodative responses. Although standardized measurement procedures and repeated readings were adopted to minimize random error, residual accommodation cannot be completely excluded. Therefore, the reported myopia prevalence, especially among primary school students in lower grades, must be interpreted with extreme caution as it may include false-positive cases. Future studies incorporating cycloplegic refraction are warranted to provide more accurate estimates of refractive status. Finally, due to the retrospective nature of the screening dataset, primary administrative logs, such as total school enrollment or reasons for missing data like absenteeism, were unavailable. As the provided dataset exclusively contained records of students with successful measurements, we were unable to calculate exact participation rates or perform a formal missing data analysis. While this precludes us from fully ruling out potential selection bias, the massive volume of the collected records provides a robust foundation for identifying overall population-level trends.

Overall, the prevalence of myopia among adolescents continues to rise, with notable disparities observed across gender, educational stage, region, and age group. Key countermeasures include strengthening vision screening and health education during primary school to cultivate healthy eye-use habits; promoting at least 2 h of daily outdoor activities; addressing urban–rural resource disparities through enhanced myopia prevention efforts in rural areas; and raising awareness among parents, teachers, and students via science-based eye health education alongside reduced digital device usage. In Qinzhou, ongoing local efforts have included mobile vision screening programs and outreach-based eye health education delivered by medical teams in rural schools and communities. These initiatives may help improve access to basic eye care services and strengthen myopia prevention awareness in rural and township areas. Future work should further improve the continuity of mobile screening, strengthen follow-up and referral after screening, and promote school- and family-based outdoor activity programs. Through these collective efforts, it is anticipated that myopia progression will be mitigated, thereby improving adolescent visual health outcomes.

## Data Availability

The original contributions presented in the study are included in the article/supplementary material, further inquiries can be directed to the corresponding authors.
